# Quaking–cZFP609 Axis Remedies Aberrant Plasticity of Vascular Smooth Muscle Cells via Mediating Platelet‐Derived Growth Factor Receptor β Degradation

**DOI:** 10.1002/mco2.70167

**Published:** 2025-04-16

**Authors:** Yong‐Qing Dou, Xiao‐Yun Zhang, Rui‐Juan Guo, Xiao‐Fu Huang, Yu Song, Xin‐Long Liu, Jie Shi, Fan‐Qin Li, Dan‐Dan Zhang, Peng Kong, Lei Nie, Han Li, Fan Zhang, Mei Han

**Affiliations:** ^1^ Department of Biochemistry and Molecular Biology College of Basic Medicine Shijiazhuang China; ^2^ College of Integrative Medicine Hebei University of Chinese Medicine Shijiazhuang China; ^3^ Key Laboratory of Neural and Vascular Biology of Ministry of Education Shijiazhuang China; ^4^ Key Laboratory of Vascular Biology of Hebei Province Hebei Medical University Shijiazhuang China; ^5^ Department of Orthopaedic Surgery Institute of Biomechanical Science and Biomechanical Key Laboratory of Hebei Province Third Hospital of Hebei Medical University Shijiazhuang China

**Keywords:** cZFP609, neointimal hyperplasia, PDGFRβ trafficking, quaking, vascular smooth muscle cells

## Abstract

Vascular smooth muscle cell (VSMC) plasticity is crucial for the repair after vascular injury. However, the high plasticity of VSMCs may make them transform into pathogenic phenotypes. Here, we show that VSMCs overexpressing Sirtuin 1 (SIRT1) exhibit a reduced phenotypic plasticity in the context of platelet‐derived growth factor (PDGF)‐BB treatment. SIRT1 activated Quaking (QKI)–cZFP609 axis is involved in the plasticity regulation in the VSMCs. Mechanically, SIRT1 deacetylates K133 and K134 of QKI and mediates its activation. Activated QKI binds the QKI response elements located in the upstream and downstream of the cZFP609‐forming exons in ZFP609 pre‐mRNA to mediate cZFP609 production. Furthermore, the acetylation of QKI is increased by inhibiting SIRT1 with the selective and potent inhibitor EX527 or deletion of SIRT1, accompanied with parallel decrease in cZFP609 formation. Final, we identify that cZFP609 directs PDGF receptor (PDGFR)β sorting into endosomal/lysosomal pathway and degradation by bridging PDGFRβ and Rab7, resulted in attenuating Raf–MEK–ERK cascade activation downstream of PDGFRβ signaling. Overexpression of cZFP609 remedies aberrant plasticity and overproliferation of VSMCs, and ameliorates neointimal formation. Together, these results highlight that modulating the QKI–cZFP609 axis may help propel repair without stenosis as a therapeutic strategy in vascular injury.

## Graphical abstract

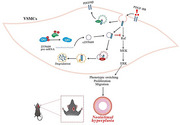

## 1 Introduction

Vascular smooth muscle cells (VSMCs), a main cell type in arterial blood vessels, contribute to vascular physiological function and vascular remodeling in various ways. The increasing evidence based on single‐cell resolution transcriptomics (scRNA‐seq) and fate‐mapping analysis demonstrate an extraordinary degree of VSMC heterogeneity and plasticity within the health arteries and atherosclerotic lesion [1, 2]. VSMCs are able to switch their phenotype from contractile to synthetic state in the context of vascular injury, accompanied with proliferation, migration, and increased extracellular matrix production, contributing to the repair process of vascular injury [3]. When the mechanisms of controlling phenotype switching fail, aberrant VSMC plasticity may increase in their susceptibility to harmful environmental cues, thus transforming them into pathogenic phenotypes [4], which not only leads to pathological over‐reparation conditions [5], but also affect the behavior of other cell types in the vessel wall [6]. However, the regulation of VSMC plasticity during vascular injury and repair is not decoded.

SIRT1, a NAD^+^‐dependent deacetylases, is a member of the mammalian sirtuin family. It has been known that SIRT1 protects against injury‐induced neointimal formation and inhibits atherosclerotic plaque progression via regulating phenotypic switching, proliferation and apoptosis of VSMCs [7, 8, 9]. SIRT1 can deacetylate many protein substrates, including transcription factors, histones, signaling components, cytoskeleton proteins, suggesting that SIRT1 participates in cardiovascular protection via a signaling network [10]. Our recent study suggested that the overexpression of SIRT1 attenuated VSMC phenotypic switching, which reduced hypoxia‐induced arteriole formation [11]. Intriguingly, *SIRT1*‐Tg VSMCs released exosome cZFP609. Furthermore, cZFP609 inhibits endothelial angiogenesis after ischemia via blockade of hypoxia‐inducible factor (HIF) 1α activated vascular endothelial growth factor expression. Noncoding circular RNAs (circRNAs), as new regulators of VSMCs, have recently been demonstrated to be implicated in the pathogenesis of vascular diseases [12]. These findings pumped us to investigate whether cZFP609 is produced by a SIRT1‐dependent pathway and contributes to reduced plasticity of VSMCs.

RNA binding proteins (RBPs) participate in regulation of VSMC functions as well as development of cardiovascular diseases [13]. The RBPs can function as splicing factors to regulate circRNA production. Quaking (QKI) is a protein of the signal transduction and activation of RNA family, which regulates pre‐mRNA splicing and circRNA production via binding to sites flanking circRNA‐forming exons during epithelial to mesenchymal transition [14]. QKI is preferentially expressed in developing vascular and heart tissues, and Qki mutant results in cardiovascular defects [15], suggesting that QKI is necessary for cell differentiation and homeostasis [16]. Compared with healthy arteries, QKI expression is upregulated in the VSMCs of human restenotic lesions [17], and the RBP is discovered to be localized at inflammatory foci [17] and to mediate dedifferentiation of VSMC via mediating alternative splicing of myocardin, a key transcription factor regulating VSMC differentiation [17]. Yet another study suggested that QKI‐6 contributes to differentiation of induced pluripotent stem cells to VSMC via mediating HDAC7 splicing and SMCs overexpressing QKI‐6 display an increased contractile ability [18]. Collectively, these studies have reinforced that QKI is critical for regulating VSMC plasticity during restenotic development.

Herein, we demonstrated that the QKI–cZFP609 axis is the key molecular pathway for the regulation of VSMC plasticity. SIRT1 activates QKI by deacetylation at K133 and K134, which mediates cZFP609 formation. The cZFP609 promotes the degradation of platelet‐derived growth factor receptors (PDGFR) β via endosomal/lysosomal pathway. QKI–cZFP609 axis confers the adaptive phenotype plasticity of VSMCs and promotes repair of vascular injury without stenosis by attenuation of PDGFRβ signaling.

## 2 Results

### 2.1 SIRT1 Reduces the Phenotypic Plasticity of VSMCs

**FIGURE 1:**
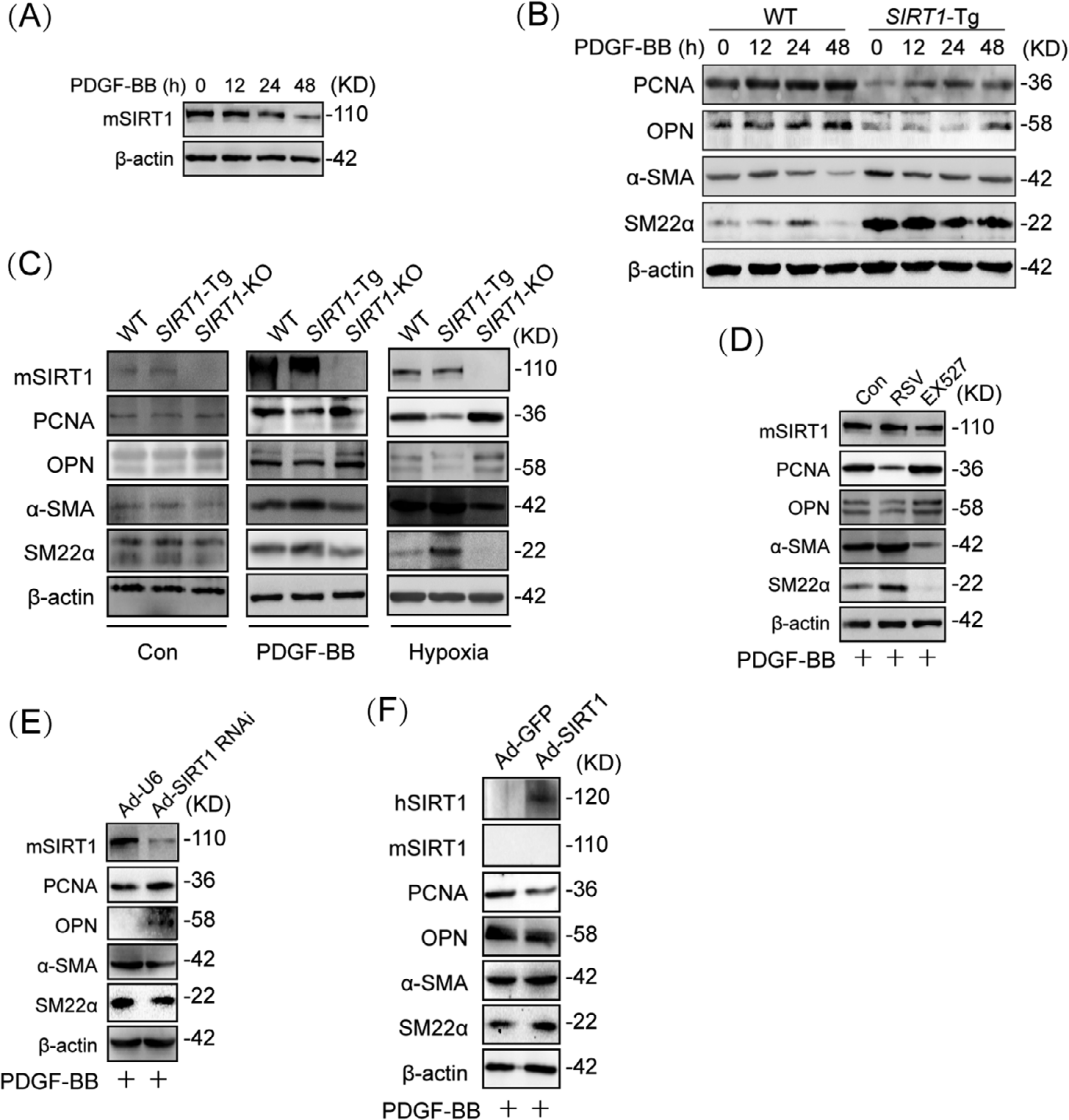
SIRT1 inhibits phenotypic switching of VSMCs. (A) Western blot for SIRT1 in WT VSMCs exposed to PDGF‐BB (10 ng/mL) for the indicated time. (B–F) Western blot of PCNA, OPN, α‐SMA, SM22α, and mSIRT1, hSIRT1. (B) In the VSMCs exposed to PDGF‐BB for 0, 12, 24, 48 h. (C) In indicated VSMCs exposed to PDGF‐BB or under hypoxia for 24 h. Before exposed to PDGF‐BB for 24 h, WT VSMCs were pretreated with DMSO, RSV, or EX527 for 4 h (D); *SIRT1‐*Tg VSMCs were infected by Ad‐U6 or Ad‐SIRT1 RNAi for 24 h (E) or *SIRT1*‐KO VSMCs were infected by Ad‐GFP or Ad‐SIRT1 for 24 h (F). *Abbreviations*: SIRT1, sirtuin 1; VSMCs, vascular smooth muscle cells; WT, wild type; KO, knockout; Tg, transgenic; PDGF‐BB, platelet‐derived growth factor‐BB; PCNA, proliferating cell nuclear antigen; OPN, osteopontin; α‐SMA, smooth muscle α‐actin; SM22α, smooth muscle 22α; RSV, resveratrol.

Since overexpression of SIRT1 inhibits VSMC proliferation and migration [7], we first examine the relationship between the expression of SIRT1 and the plasticity of VSMCs. The expression of SIRT1 was reduced in a time‐dependent manner in mouse VSMCs upon PDGF‐BB treatment (Figures 1A and S1A). To examine the effect of SIRT1 on VSMC plasticity, VSMCs of (*SIRT1*‐Tg) mice were treated with PDGF‐BB. The expression of endogenous SIRT1 was not significantly changed in *SIRT1*‐Tg VSMCs upon PDGF‐BB treatment (Figure S1B), which exhibited higher expression of α‐SMA (smooth muscle α‐actin) and SM22α, and a slightly increased level of PCNA (proliferating cell nuclear antigen) and OPN (osteopontin), compared with wild‐type (WT) VSMCs, as indicated by Western blot (Figures 1B and S1C), suggesting that the overexpression of SIRT1 impaired the phenotypic plasticity of VSMCs. In contrast, SIRT1 deletion promoted the phenotypic switching of VSMCs, which exhibited higher PCNA and OPN level, and lower expression of α‐SMA and SM22α than that of WT cells induced by PDGF‐BB (Figures 1C and S1D). Similarly, hypoxia‐induced phenotypic switching was inhibited in *SIRT1*‐Tg VSMCs, and enhanced in *SIRT1*‐KO VSMCs (Figures 1C and S1D), compared with WT cells (Figure S1E). To further confirm the role of SIRT1 in regulating VSMC plasticity, VSMCs were pretreated with pharmacological agents that activate or inhibit SIRT1 activity. Compared with no PDGF‐BB‐induced VSMCs (Figure S1F), SIRT1 agonist resveratrol (RSV) inhibited PDGF‐BB‐induced expression of synthetic markers PCNA and OPN, while SIRT1 inhibitor EX527 promoted phenotypic switching under the same conditions (Figures 1D and S1G). Furthermore, *SIRT1‐*Tg VSMCs infected with Ad‐shSIRT1 displayed an enhanced synthetic phenotype, compared with *SIRT1‐*Tg VSMCs infected with Ad‐U6 as negative control (Figures 1E and S1H). Conversely, rescue of SIRT1 expression in *SIRT1*‐KO VSMCs by infection of Ad‐SIRT1 abolished PDGF‐BB‐induced phenotypic switching, compared with Ad‐GFP group (Figures 1F and S1I). Collectively, these data suggest that SIRT1 abates the plasticity of VSMCs in response to PDGF‐BB.

### 2.2 SIRT1 Promoting cZFP609 Expression Results in a Reduced Plasticity of VSMCs

**FIGURE 2:**
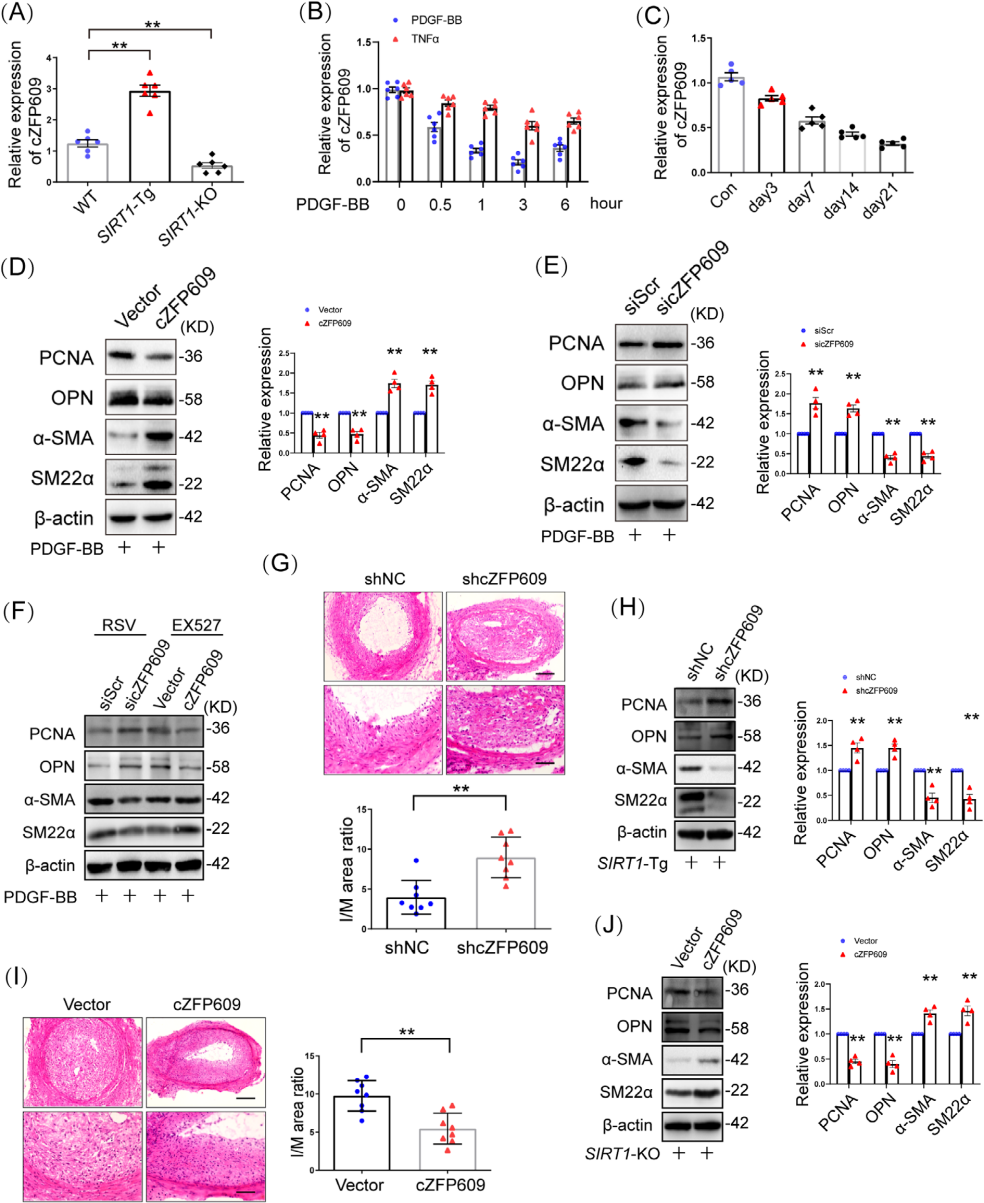
cZFP609 mediates the inhibitory effect of SIRT1 on phenotypic switching of VSMCs. (A–C) qRT‐PCRs of cZFP609 expression. (D–F, H, J) Western blot of PCNA, OPN, α‐SMA, SM22α. (A) In carotid arteries of mice. (B) WT VSMCs exposed to PDGF‐BB or TNFα for indicated time. (C) The carotid arteries were ligated for the indicated time in WT mice. (D) WT VSMCs were infected by plasmid cZFP609 for 24 h, then exposed to PDGF‐BB for 24 h. (E) WT VSMCs were infected by sicZFP609 for 24 h, then exposed to PDGF‐BB for 24 h. (F) WT VSMCs were transfected by cZFP609 plasmid or sicZFP609 for 20 h, pretreated by RSV or EX527 for 4 h, then exposed to PDGF‐BB for 24 h. (G and H) AAV9–shcZFP609 (1 × 10^12^ vg/mL, 100 µL per mouse) was injected into *SIRT1*‐Tg mice by tail vein for 35 days, then carotid artery ligation for 14 days. (G) Representative histological sections and the intima to media (I/M) ratio of carotid arteries. Upper scale bars = 100 µm, lower scale bars = 50 µm (*n* = 8). (H) In ligated carotid arteries. (I and J) AAV9–cZFP609 (1 × 10^12^ vg/mL, 100 µL per mouse) was injected into *SIRT1*‐KO mice by tail vein for 35 days, then carotid artery ligation for 28 days. (I) Representative histological sections and the intima to media (I/M) ratio of carotid arteries. Upper scale bar = 100 µm, lower scale bar = 50 µm (*n* = 8), (J) In ligated carotid arteries. Date are presented as mean ± SEM. Student's *t*‐test or one‐way ANOVA: *p < 0.05, **p < 0.01 versus the corresponding control.

We have demonstrated that smooth muscle SIRT1 inhibits ischemia‐induced angiogenesis of endothelial cells via exosome cZFP609 [11]. To determine whether cZFP609 mediates SIRT1 inhibiting VSMC plasticity, we first examined the relationship between SIRT1 and cZFP609 formation in VSMCs and showed that cZFP609 formation was increased in *SIRT1*‐Tg VSMCs and reduced in VSMCs with *SIRT1*‐KO compared with that in WT cells (Figure S2A). Activation of SIRT1 by RSV increased cZFP609 formation, and conversely, EX527, an inhibitor of SIRT1, reduced it (Figure S2B). Similarly, EX527 also abolished increased cZFP609 formation in *SIRT1*‐Tg VSMCs (Figure S2C). Furthermore, we found that the production of cZFP609 was higher in the artery of *SIRT1*‐Tg than that of WT mice (Figure 2A). These data suggest that SIRT1 contributes to cZFP609 production.

To determine the profile of cZFP609 formation during VSMC phenotypic switching, VSMCs was treated with PDGF‐BB. We showed that the level of cZFP609 reduced in a time‐dependent manner upon either PDGF‐BB or TNFα treatment (Figure 2B) and in the neointima formation in vivo, compared with the sham control (Figure 2C).

To check the effect of cZFP609 on VSMC phenotypic plasticity, cZFP609 was overexpressed in VSMCs. Overexpression of cZFP609 inhibited the expression of PCNA and OPN and elevated the levels of α‐SMA and SM22α in VSMCs in the context of PDGF‐BB treatment (Figures 2D and S2D), accompanied by reduced proliferation and migration of VSMCs (Figure S2E,F). In contrast, the knockdown of cZFP609 enhanced phenotypic switching (Figures 2E and S2G), proliferation and migration of VSMCs (Figure S2H,I), indicating that cZFP609 diminishes the plasticity of VSMCs. To assess whether cZFP609 mediates SIRT1 reducing phenotypic plasticity of VSMCs, *SIRT1*‐Tg VSMCs were transfected with the specific small interfering RNA (siRNA) to silence cZFP609 expression. The phenotypic switching was induced in *SIRT1*‐Tg VSMCs with cZFP609 silencing upon PDGF‐BB treatment (Figure S2J), accompanied by increased proliferation and migration (Figure S2K,L). Consistently, reduced VSMC plasticity by activating SIRT1 was abolished by cZFP609 silencing, and enhanced by overexpression of cZFP609 in VSMC (Figures 2F and S2M).

It has been demonstrated that SIRT1 inhibits neointimal hyperplasia in mice [7]. To determine the role of cZFP609 in this progress, an adeno‐associated virus serotype 9 (AAV9)–shcZFP609 was transfered into *SIRT1*‐Tg mice by tail vein injection before the carotid artery ligation. We showed that the production of cZFP609 was decreased by 80% in the carotid arteries of *SIRT1*‐Tg mice administrated with AAV9–shcZFP609 (Figure S2N), suggesting that a higher efficiency of cZFP609 knockdown is achieved by AAV9–shRNA in vivo. The neointimal hyperplasia in the AAV9–shcZFP609‐administrated mice was aggravated, which displayed higher the ratio of intima and media area (I/M) compared with the AAV9–shNC group (Figure 2G). Of course, higher expression of VSMC contractile markers was abrogated in *SIRT1*‐Tg mice with cZFP609 silencing, accompanied by increase in the synthetic markers (Figure 2H).

In line with the above observation, compared with the vehicle, overexpression of cZFP609 significantly retarded the neointimal hyperplasia in the *SIRT1‐*KO mice (Figures S2O and 2I). The I/M ratio was decreased with increased cZFP609 expression (Figure 2I) in carotid arteries, accompanied with increased contractile gene expression and decreased synthetic markers (Figure 2J). Overall, these results suggest that cZFP609 attenuates VSMC phenotypic plasticity and neointimal hyperplasia.

### 2.3 QKI is Responsible for cZFP609 Formation in VSMCs

**FIGURE 3:**
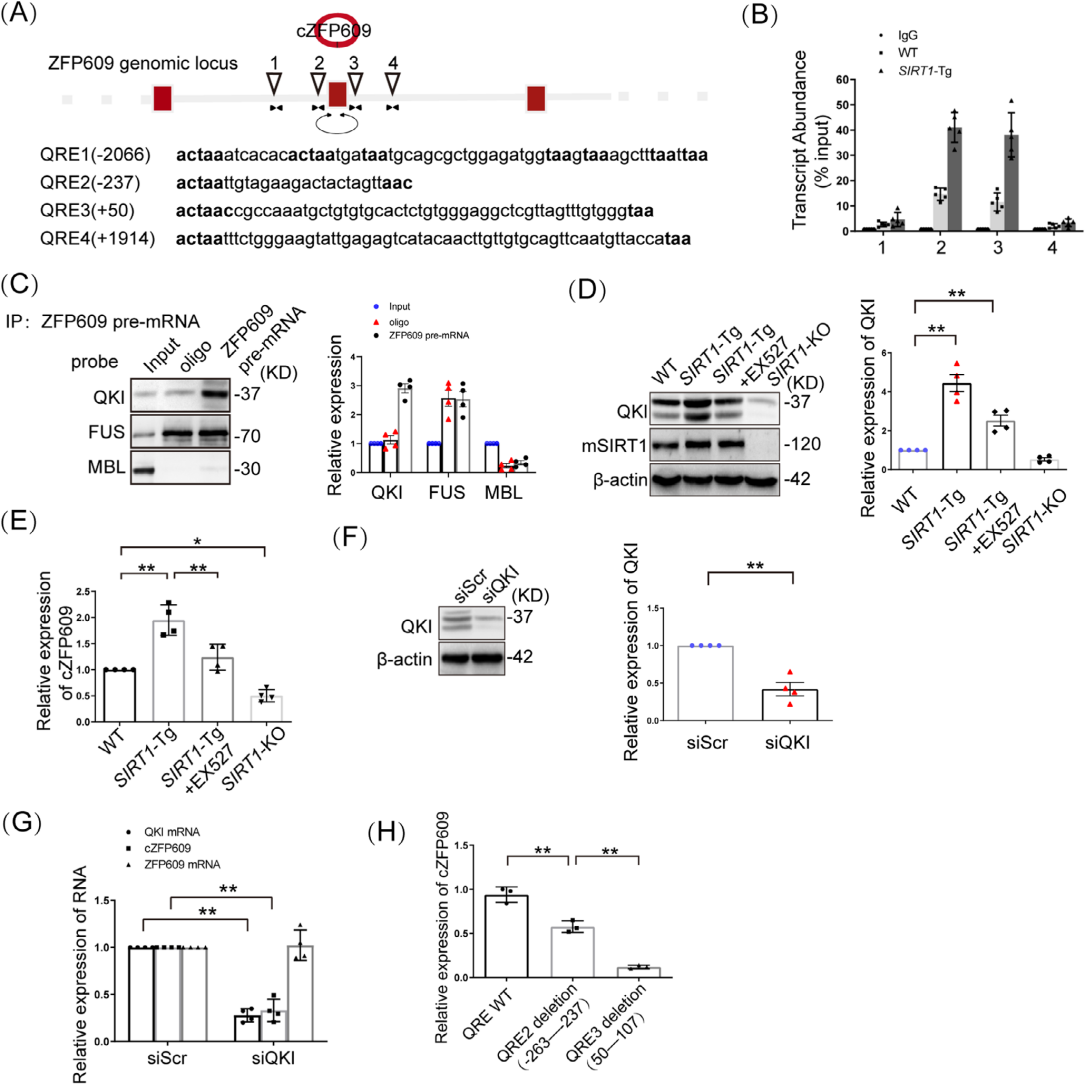
QKI is responsible for formation of cZFP609 in *SIRT1*‐Tg VSMCs. (A) Schematic of ZFP609 pre‐mRNA showing the locations of four putative QREs (inverted triangles) and amplicons used for RIP assay. (B) RIP assay was performed using QKI antibodies in the VSMCs and using the PCR primers indicated in (A). (C) RNA pull‐down assay was performed using the probes for the introns flanking the circRNA‐forming exons (approximately 500 bp upstream and downstream) of ZFP609 in the *SIRT1‐*Tg VSMCs. Western blot was used to validate the interactions between ZFP609 pre‐mRNA and alternative splicing factors. (D, E, and G) The cells were exposed to PDGF‐BB for 24 h. (D and E) VSMCs were pretreated with DMSO or EX527 for 4 h and then exposed to PDGF‐BB. (D) Western blot analysis of QKI and SIRT1. (E and H) qRT‐PCR of cZFP609 expression. (F and G) The *SIRT1*‐Tg VSMCs were transfected with siScr or siQKI for 24 h and then exposed to PDGF‐BB for 24 h. (F) QKI protein expression was detected by Western blot. (G) qRT‐PCR of cZFP609, QKI, and ZFP609 mRNA expression. (H) *SIRT1*‐Tg VSMCs were transfected by recombinant ZFP609 mutant, QRE WT, the deletion mutant of putative QRE2 and 3 for 48 h. Bar graphs show mean ± SEM values from three independent experiments (*n* = 3). Student's *t*‐test or one‐way ANOVA: *p < 0.05, **p < 0.01 versus the corresponding control. *Abbreviations*: QKI, Quaking; QRE, QKI response element; IP, immunoprecipitation.

To elucidate the mechanism underlying cZFP609 production in *SIRT1*‐Tg VSMCs, we performed multiple sequence alignment using a bioinformatics approach as previously described [19, 20] and found putative muscleblind (MBL) binding sites and the four QKI response elements (QRE) located in the introns flanking exons forming cZFP609 splice sites (Figure 3A). To validate that QKI binds ZFP609 pre‐mRNA, RNA‐immunoprecipitation (RIP) assays were conducted to quantify QKI occupancy within the introns adjacent to cZFP609‐forming exons. We showed that QKI bound to the exon‐adjacent QRE sites, which was increased in *SIRT1*‐Tg VSMCs (Figure 3B), whereas binding to more remote QRE sites elsewhere in ZFP609 was negligible (Figure 3B). Consistently, using RNA pull‐down, we verified that the interaction of QKI with the introns flanking the circRNA‐forming exons displayed a stronger affinity than with MBL (Figure 3C). However, FUS binding to Alu elements revealed nonspecific binding under the same conditions. Moreover, QKI protein was highly expressed in *SIRT1*‐Tg VSMCs, and reduced following inhibition or knockout of SIRT1 (Figure 3D), accompanied by a parallel shift in cZFP609 level (Figure 3E).

To confirm that QKI mediates cZFP609 production, the knockdown of QKI was conducted in *SIRT1*‐Tg VSMCs. QKI silencing diminished circular product, and not changed linear product (Figure 3F,G). To verify that QREs located in the introns flanking cZFP609‐forming exons is required for cZFP609 formation, *SIRT1*‐Tg VSMCs were infected with the deletion mutant of putative QRE2 or QRE3, and consequently, cZFP609 production was repressed compared with WT QRE that increased cZFP609 formation (Figure 3H). Collectively, these data suggest that QKI contributes to cZFP609 formation.

### 2.4 SIRT1 deacetylates and Activates QKI

**FIGURE 4:**
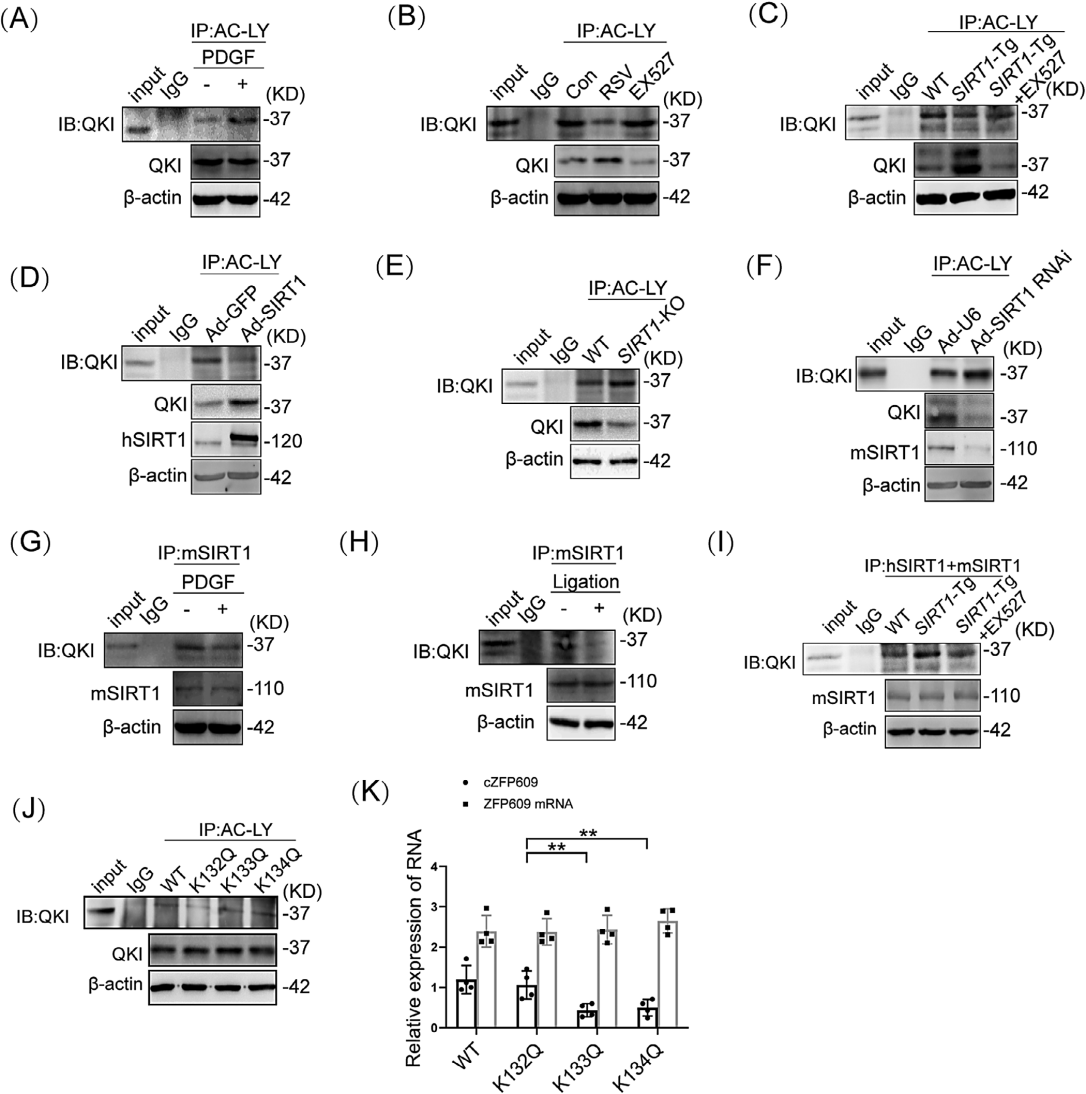
SIRT1 deacetylates and activates QKI. (A–F) Immunoprecipitation and Western blot analysis for QKI acetylation. (A) WT VSMCs were exposed to PDGF‐BB for 24 h. (B and C) WT (B) or *SIRT1*‐Tg VSMCs (C) from mice were pretreated with RSV or EX527 for 4 h. (D) WT VSMCs were infected by Ad‐GFP or Ad‐SIRT1. (E) WT and *SIRT1*‐KO VSMCs. (F) *SIRT1*‐Tg VSMCs were infected by Ad‐U6 or Ad‐SIRT1 RNAi. (G–I) Coimmunoprecipitation for the interaction between SIRT1 and QKI in mouse VSMCs. (G) WT VSMCs were exposed to PDGF‐BB for 24 h. (H) Nonligated and ligated carotid arteries for 21 days in WT mice. (I) WT or *SIRT1*‐Tg VSMCs were pretreated with EX527 for 4 h. J‐K *SIRT1*‐Tg VSMCs were infected by recombinant QKI mutant, WT, K132Q, K133Q, and K134Q, for 48 h. (J) Immunoprecipitation and Western blot analysis for QKI acetylation. (K) qRT‐PCR of expression of cZFP609 and ZFP609 mRNA. Bar graphs show mean ± SEM values from four independent experiments (*n* = 4). Student's *t*‐test or one‐way ANOVA: *p < 0.05, **p < 0.01 versus the corresponding control. *Abbreviation*: IB, Western blot.

It has been known that the activity of QKI is regulated by posttranslation modification [21] and that SIRT1 makes QKI 5 deacetylation to regulate cellular functions [22]. Using the pan‐acetyl‐Lys antibody, we found that the level of QKI acetylation was elevated in VSMCs upon PDGF‐BB treatment (Figure 4A). To validate that SIRT1 deacetylates QKI, we next examined the relationship between SIRT1 expression and activity and QKI acetylation. The activation of SIRT1 by RSV decreased the acetylation of QKI, while inhibition of SIRT1 by EX527 increased QKI acetylation (Figure 4B). This was confirmed by performing Western blot on immunoprecipitation with pan‐acetyl‐Lys antibody in RSV‐ or EX527‐induced VSMCs. Consistent with a role for SIRT1 activation or inhibition in regulating QKI acetylation, the acetylation of QKI decreased in *SIRT1*‐Tg or SIRT1‐overexpressed VSMCs (Figure 4C,D) and increased in *SIRT1*‐KO or knockdown of SIRT1 cells (Figure 4E,F), compared with the control under the same conditions. Next, the interaction between SIRT1 and QKI was validated using co‐immunoprecipitation (co‐IP) analysis. QKI was co‐immunoprecipitated by anti‐SIRT1 antibody but not by control IgG in VSMCs, which was markedly decreased in PDGF‐BB‐induced VSMCs (Figure 4G), parallel with increased QKI acetylation. Consistently, co‐IP analysis for the normal artery lysates of mice further demonstrated the interaction between SIRT1 and QKI in vivo (Figure 4H), which was reduced in the neointimal hyperplasia. Notably, the interaction of SIRT1 with QKI was enhanced in *SIRT1*‐Tg VSMCs and abrogated by inhibiting SIRT1 with EX527 (Figure 4I), consistent with decreased and increased QKI acetylation under the same conditions. Together, these results indicate that SIRT1 may mediate QKI deacetylation.

To further confirm QKI deacetylation regulated by SIRT1, we predicted the potential acetylation site of QKI through ASEB (http://bioinfo.bjmu.edu.cn/huac/predict_p/), and selected three lysine sites (*p* < 0.05) to construct Ac‐mimic mutants (mutation from K to Q). *SIRT1*‐Tg VSMCs were infected by the Ac‐mimic mutants (K132Q, K133Q, and K134Q) of QKI, respectively. Decreased acetylation of QKI was observed in *SIRT1*‐Tg VSMCs infected by the mutants of WT and K132Q but not K133Q and K134Q mutants by the pan‐acetyl‐Lys antibody (Figure 4J). Meantime, the formation of cZFP609 but not linear product was decreased in *SIRT1*‐Tg VSMCs infected by K133Q and K134Q mutants and increased in the K132Q‐infected cells (Figure 4K). Overall, these data suggest that K133 and K134 are the major sites of QKI deacetylation and activation.

### 2.5 cZFP609 Blocks the Raf–MEK–ERK Signaling in VSMCs

**FIGURE 5:**
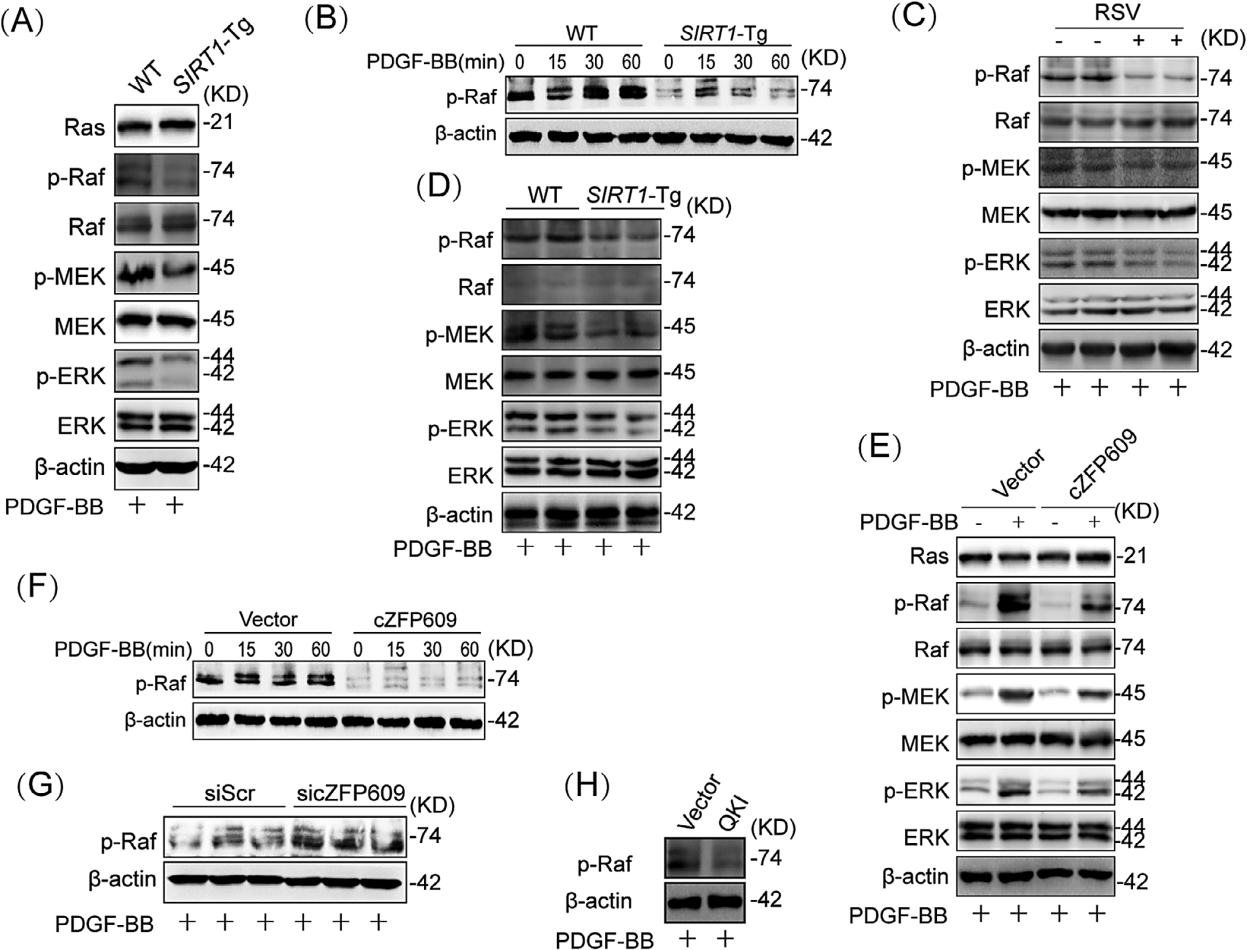
SIRT1 inhibits the Raf–MEK–ERK signaling by cZFP609 in VSMCs. (A, C, D, and E) Western blot of the levels of total and phosphorylated Raf, MEK, ERK, and Ras indicated proteins, (A, C, D, E, G, and H) The VSMCs exposed to PDGF‐BB for 30 min. (B, F–H) Western blot of phosphorylated Raf. (B) The VSMCs were stimulated with PDGF‐BB for indicated time. (C) Before exposed to PDGF‐BB, WT VSMCs were pretreated with RSV for 4 h. (D) The carotid arteries ligated for 21 days in WT and *SIRT1*‐Tg mice. (E, G, and H) The VSMCs infected by the indicated vectors or siRNA for 48 h, then stimulation with PDGF‐BB for 30 min. (E) WT VSMCs. (F) *SIRT1*‐KO VSMCs infected by the indicated vectors for 48 h, then stimulation with PDGF‐BB for indicated time. (G) *SIRT1*‐Tg VSMCs, (H) WT VSMCs. *Abbreviations*: Raf, rapidly accelerated fibrosarcoma; MEK, MAPK/ERK kinase; ERK, extracellular‐signal‐regulated kinase.

To investigate how cZFP609 attenuated VSMC phenotypic switching induced by PDGF‐BB, we conducted proteomics on contractile and PDGF‐BB‐induced synthetic VSMCs. KEGG pathway analysis revealed that the differential expressed proteins were recruited in lysosome, endocytosis, and MAPK signaling pathways (Figure S3). We and others have demonstrated that PDGF‐BB‐activated Raf–MEK–ERK MAPK signaling induces the phenotypic switching and proliferation of cells [23, 24]. We hypothesized that SIRT1‐mediated cZFP609 formation suppresses Raf–MEK–ERK–MAPK pathway activation. We first assessed the inhibitory effect of SIRT1 on PDGF‐BB activated Raf–MEK–ERK signaling cascades, and showed a robust reduced phosphorylation of Raf, MEK, and ERK in *SIRT1*‐Tg VSMCs compared with the WT cells, as determined by using kinase phosphorylation assays (Figure 5A,B). However, overexpression of SIRT1 did not show a markedly difference in Ras expression (Figure 5A), implying that PDGF‐BB signaling was terminated at Raf by SIRT1. Furthermore, the activation of SIRT1 by RSV circumvented PDGF‐BB‐induced phosphorylation of Raf–MEK–ERK (Figure 5C). In accordance, the neointima of *SIRT1*‐Tg mice also exhibited reduced phosphorylation of Raf–MEK–ERK (Figure 5D).

To validate whether cZFP609 is involved in this inhibitory effect of SIRT1, cZFP609 plasmid was transfected into VSMCs. As expected, overexpression of cZFP609 decreased phosphorylation of Raf, MEK, and ERK upon PDGF‐BB stimulation, compared with VSMCs transfected with the vehicle (Figure 5E). Similarly, overexpression of cZFP609 abrogated phosphorylation of Raf induced by PDGF‐BB in *SIRT1*‐KO VSMCs (Figure 5F). In contrast, knockdown of cZFP609 in *SIRT1*‐Tg VSMCs rescued the phosphorylation of Raf (Figure 5G). Of course, overexpression of QKI also induced a similar effect to cZFP609, which decreased the level of Raf phosphorylation (Figure 5H). Together, these data suggest that SIRT1 inhibits the Raf–MEK–ERK signaling by cZFP609 in VSMCs.

### 2.6 Overexpression of cZFP609 Reduces PDGFRβ Protein Expression in VSMCs in Vivo and in Vitro

**FIGURE 6:**
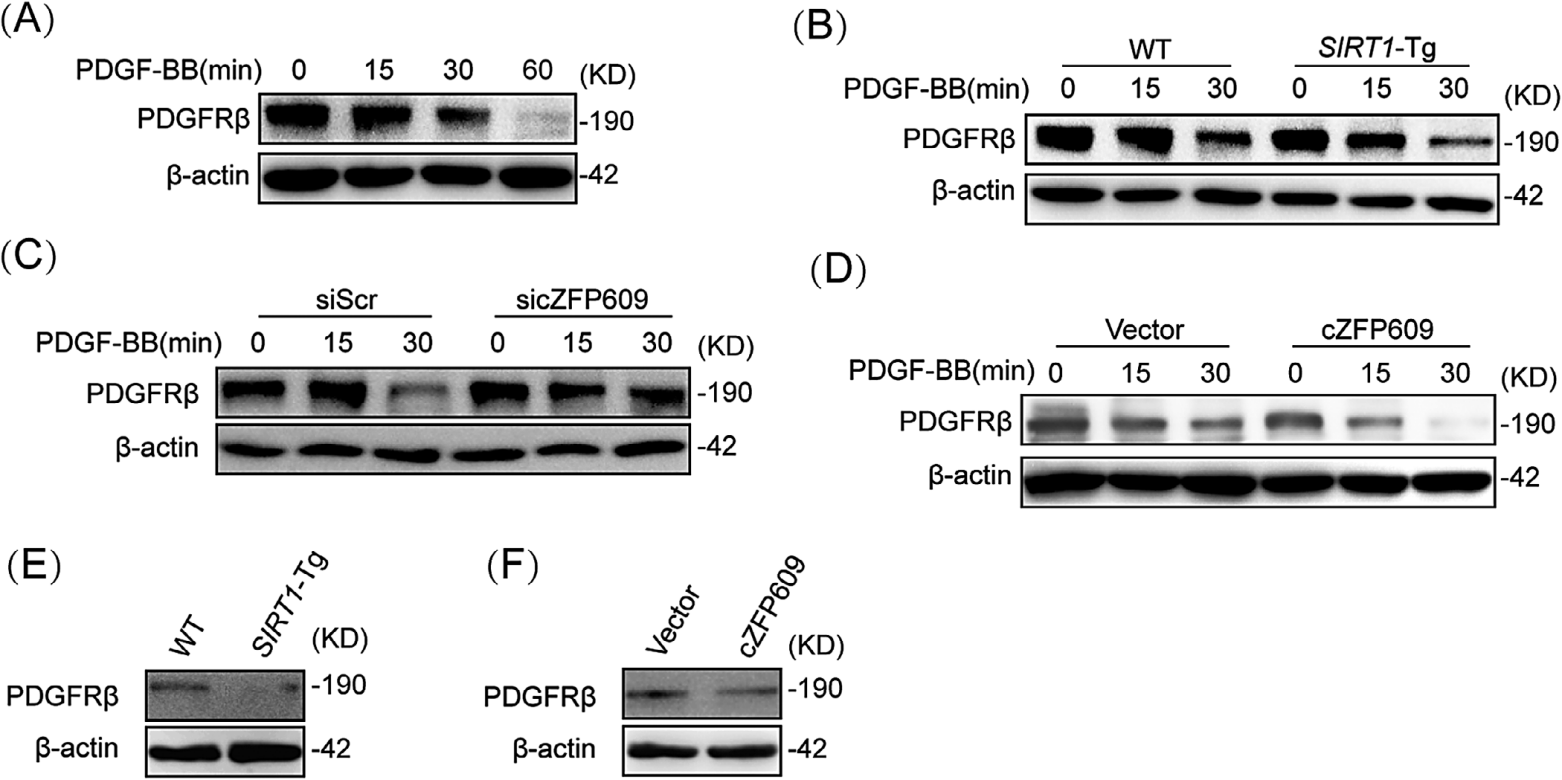
Overexpression of cZFP609 reduces PDGFRβ protein level in VSMCs in vivo and in vitro. (A–F) Western blot of PDGFRβ. WT VSMCs (A), WT and *SIRT1*‐Tg VSMCs (B), *SIRT1*‐Tg VSMCs transfected with siScr or sicZFP609 for 24 h (C) or *SIRT1*‐KO VSMCs transfected by the indicated vectors for 48 h (D) and then exposed to PDGF‐BB for the indicated time. (E and F) The mice carotid arteries were ligated for 21 days. Western blot for expression of PDGFRβ in ligated carotid arteries of mice. (E) WT and *SIRT1*‐Tg mice. (F) Before carotid artery ligation, AAV9–cZFP609 (1 × 10^12^ vg/mL, 100 µL per mouse) was injected into WT mice by tail vein for 35 days. *Abbreviation*: PDGFRβ, PDGF receptor β.

It has been demonstrated that PDGFRβ expression is increased in neointimal hyperplasia, which has been implicated in vascular remodeling via activation of its downstream signaling MAPKs [25]. We showed that the level of PDGFRβ protein decreased in WT VSMCs after PDGF‐BB treatment for 60 min (Figure 6A), and lower in *SIRT1*‐Tg VSMCs than that in WT VSMCs at 30 min upon PDGF‐BB treatment (Figure 6B). Notably, knockdown of cZFP609 increased PDGFRβ expression in *SIRT1*‐Tg VSMCs (Figure 6C). In accordance, overexpression of cZFP609 markedly decreased PDGFRβ levels in *SIRT1*‐KO VSMCs (Figure 6D). Moreover, the level of PDGFRβ protein reduced in the neointimal hyperplasia of *SIRT1*‐Tg mice, compared with WT mice (Figure 6E). A similar effect was observed in the neointima formation of WT mice overexpressing cZFP609, which exhibited a decrease in PDGFRβ protein level (Figure 6F). Therefore, PDGFRβ may be a potential target for blocking Raf–MEK–ERK signaling by cZFP609.

### 2.7 cZFP609 Promotes PDGFRβ Sorting into Endosomal/Lysosomal Components for Degradation

**FIGURE 7:**
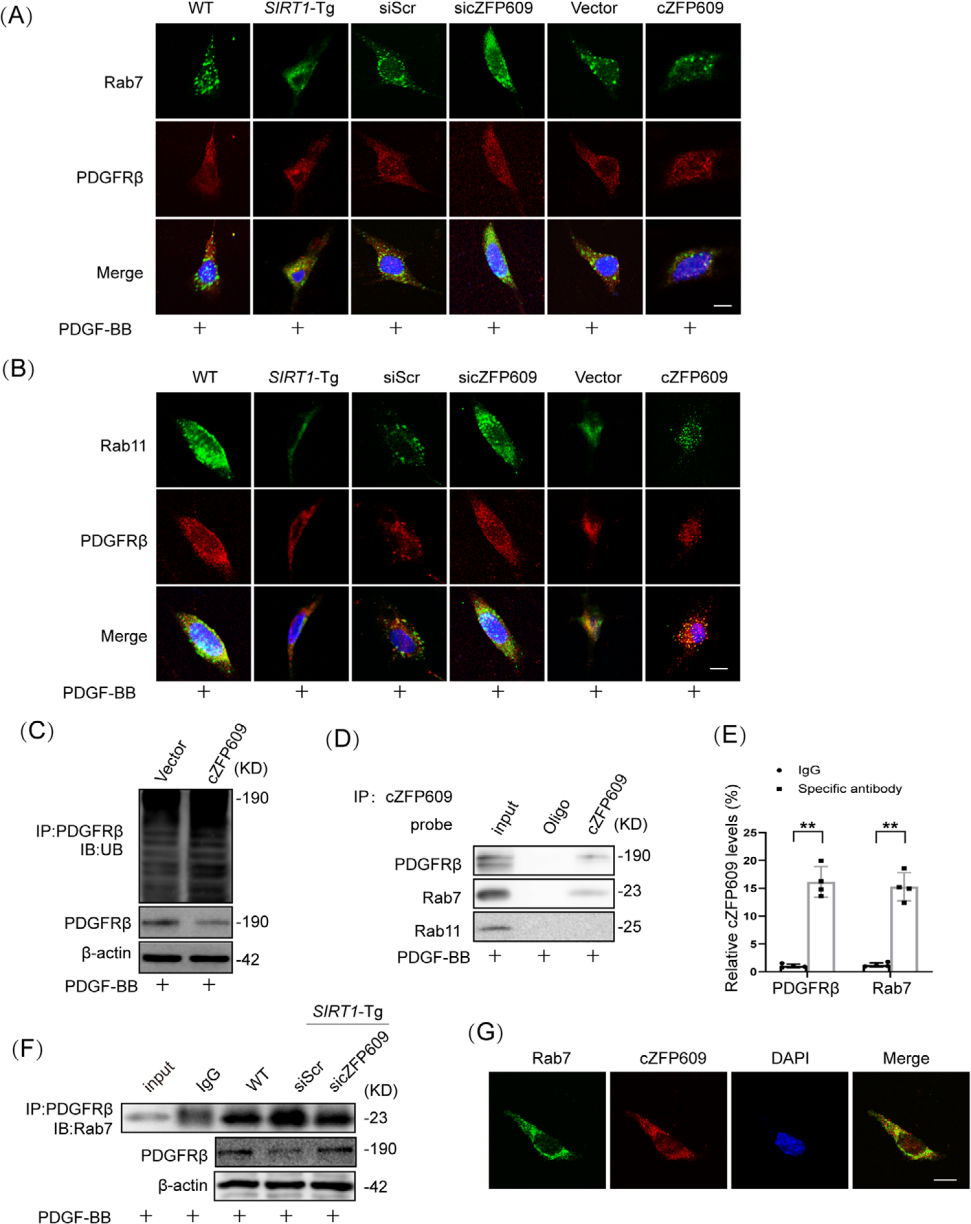
cZFP609 promotes PDGFRβ sorting into endosomal/lysosomal component to degradation. (A) Costained for internalized PDGFRβ (red) and Rab7 (green) in the VSMCs (left), *SIRT1*‐KO VSMCs transfected by the indicated vectors (middle) or *SIRT1*‐Tg VSMCs transfected with siScr or sicZFP609 (right) for 48 h and then exposed to 10 ng/mL of PDGF‐BB for 30 min. (B) Costained for internalized PDGFRβ (red) and Rab11 (green) in the VSMCs (left), *SIRT1*‐Tg VSMCs transfected with siScr or sicZFP609 (middle) or *SIRT1*‐KO VSMCs transfected by the indicated vectors for 48 h (right) and then exposed to 10 ng/mL of PDGF‐BB for 30 min. Scale bars = 10 µm. (C) WT VSMCs transfected by the indicated vectors for 48 h and then exposed to 10 ng/mL of PDGF‐BB for 30 min. Immunoprecipitation for PDGFRβ ubiquitination. (D) RNA pull‐down assay was performed using the probe. Western blot was used to validate the interactions between cZFP609 and PDGFRβ, Rab7, and Rab11 in SIRT1‐Tg VSMCs. (E) RIP assay was performed using PDGFRβ or Rab7 antibodies in SIRT1‐Tg VSMCs. qRT‐PCR was used to detect pulled‐down cZFP609. (F) Coimmunoprecipitation for the interaction between PDGFRβ and Rab7 in the VSMCs. (G) Confocal FISH images of colocalization between Rab7 and cZFP609 in the *SIRT1*‐Tg VSMCs. Scale bars = 10 µm. Bar graphs show mean ± SEM from four independent experiments (*n* = 4). Student's *t*‐test: *p < 0.05, **p < 0.01 versus the corresponding control. *Abbreviation*: UB, ubiquitin.

The PDGFR binding to the ligand causes its internalization. The internalized PDGFR either recycles back to the cell surface [26, 27] or is sorted to the lysosome for degradation [28, 29]. To determine that cZFP609 regulates the trafficking of PDGFRβ, the colocalization of PDGFRβ, and early endosomal antigen 1 (EEA‐1), an early endosome marker was detected using immunofluorescence staining. No difference in the colocalization of PDGFRβ with EEA‐1 was observed between *SIRT1*‐Tg and WT VSMCs at 15 min of PDGF‐BB treatment (Figure S4A). In accordance, knockdown of cZFP609 in *SIRT1*‐Tg VSMCs (Figure S4B) or overexpression of cZFP609 in *SIRT1*‐KO VSMCs (Figure S4C) did not change the colocalization of PDGFRβ with EEA‐1. Notably, increased colocalization of PDGFRβ with Rab7, a marker of the late endosomal/lysosomal compartment, was observed in *SIRT1*‐Tg VSMCs (Figure 7A, left) and in cZFP609‐overexpressed cells (Figure 7A, middle) at 30 min of PDGF‐BB treatment. In contrast, knockdown of cZFP609 reduced colocalization of PDGFRβ and Rab7 (Figure 7A, right), consistent with increased the level of PDGFRβ protein, speculating that cZFP609 may promote the PDGFRβ sorting into endosomal/lysosomal component to degradation.

Internalized receptors usually recycle back to the membrane surface via recycling endosomes in a Rab11‐dependent manner [30]. To validate our speculation, we performed coimmunostaining of PDGFRβ and Rab11. In line with the above observation, colocalization of PDGFRβ and Rab11 was decreased in *SIRT1*‐Tg VSMCs (Figure 7B, left). Knockdown of cZFP609 increased colocalization of PDGFRβ and Rab11 (Figure 7B, middle), while overexpression of cZFP609 reduced colocalization of PDGFRβ with Rab11 (Figure 7B, right). Importantly, PDGFRβ ubiquitination, a potential signal for degradation [31, 32], was increased in VSMCs with overexpression of cZFP609 (Figure 7C), leading to signal attenuation.

Taken together, these results indicate that cZFP609 interrupts PDGFRβ trafficking through recycling endosomes, which leads to a reduction in phosphorylation of the downstream signal proteins, thereby, attenuating PDGFRβ signaling.

### 2.8 cZFP609 may Act as an Endosomal Sorting Signal to Direct Diverting PDGFRβ into Lysosomes

To elucidate how cZFP609 directs PDGFRβ sorting into late endosomal/lysosomal, RNA pull‐down and RIP assays were conducted to detect the interaction of cZFP609 with PDGFRβ. The lysates from *SIRT1*‐Tg VSMCs were incubated with a biotinylated DNA probe targeting cZFP609, which showed a strong interaction of cZFP609 with PDGFRβ and Rab7. Both PDGFRβ and Rab7 were pulled down by the DNA probe, and Rab11 did not so (Figure 7D). Coincidently, cZFP609 was immune‐precipitated by the antibodies against PDGFRβ and Rab7 (Figure 7E). Importantly, the interaction between PDGFRβ and Rab7 was markedly enhanced in *SIRT1‐*Tg VSMCs and reduced by knockdown of cZFP609 (Figure 7F). Fluorescence in situ hybridization (FISH) and laser confocal microscopy showed the interaction between Rab7 and cZFP609 in the VSMCs (Figure 7G). Together, these results suggest that cZFP609 directs PDGFRβ late endosomal/ lysosomal degradation by bridging PDGFRβ and Rab7.

## 3 Discussion

**FIGURE 8:**
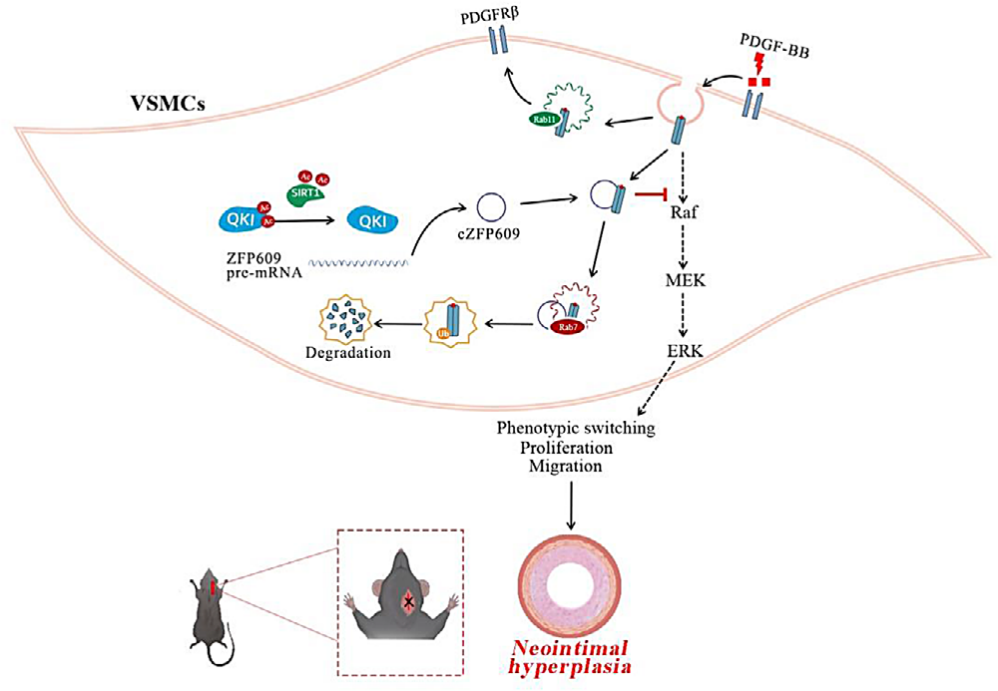
QKI–cZFP609 axis damps aberrant VSMC plasticity and mitigates vascular remodeling via directing PDGFRβ into endosomal/lysosomal degradation.

In the current study, we elucidated that QKI–cZFP609 axis recapitulated the control on the high plasticity of VSMCs. QKI and its mediated cZFP609 formation are the key molecular pathway and a new target for the regulation of VSMC plasticity and validated their protective roles in intimal hyperplasia. Deacetylation at K133 and K134 is required for QKI activation. QKI mediates cZFP609 formation via binding QRE sites upstream and downstream of the exons forming cZFP609 in ZFP609 pre‐mRNA. Furthermore, cZFP609, as a signal for sorting the transmembrane proteins into endosomes and lysosomes, dampens PDGFRβ signaling via directing it into endosomal/lysosomal degradation, contributing to finely control of VSMC plasticity and mitigating of intima hyperplasia (Figure 8).

The most known function of QKI regulates pre‐mRNAs alternative splicing [33]. QKI phosphorylation by Src decreases its binding to target RNA, reducing its ability to regulate mRNAs [34, 35]. SIRT1‐mediated QKI deacetylation is associated with the regulation of pre‐mRNA splicing and gene expression [36, 37, 38]. We demonstrated that deacetylation of QKI at K133 and K134 is required for QKI activation, contributing to cZFP609 formation. Decreased acetylation of QKI was abrogated in the VSMCs infected by the K133Q and K134Q mutants, compared with QKI WT and K132Q. Consistently, the formation of cZFP609 was decreased in the VSMCs infected by K133Q and K134Q mutants and increased in the K132Q‐infected cells, suggesting that OKI activation is dependent on K133 and K134 deacetylation.

CircRNAs are a class of noncoding regulatory RNAs [39] via acting as protein decoys, scaffolds, and recruiters to function by interacting with multiple proteins under different circumstances to cement or to dissociate interactions between proteins in diverse physiological and pathological contexts [40]. Several circRNAs, such as circANRIL, circMAP3K5, circSirt1, and circLRP6, have recently been identified in VSMCs, which regulate VSMC phenotypic switching, migration, proliferation, and inflammation [41, 42, 43, 44]. However, it is unknown about circRNA regulation on PDGFR trafficking in VSMC phenotype modulation. Herein, we identified that cZFP609 expression was significantly downregulated in mouse intimal hyperplasia and PDGF‐BB‐treated VSMCs. With evidence from a series of gain‐ and loss‐of‐function studies, we demonstrate that cZFP609 reduces VSMC phenotype plasticity by attenuating the PDGFRβ signaling through directing PDGFRβ sorting into endosomal/lysosomal component. Meantime, PDGFRβ ubiquitination and degradation were increased in VSMCs with overexpression of cZFP609 under the same conditions. We thought that cZFP609 was higher binding ability to Rab7 than that to Rab11 as shown in Figure 7D, and thereby PDGFRβ was preferentially recruited by cZFP609 to endosomal/lysosomal pathway for degradation. Thus, cZFP609 may act as an endosomal sorting signal to direct diverting PDGFRβ into lysosomes. Our results provide evidence that the circRNA is involved in endosomal sorting, which regulates PDGFRβ trafficking.

The present study has several limitations. First, we did not identify the isoform of QKI modulating VSMC plasticity as each isoform may be distributed differently in the nucleus and the cytoplasm and may differently regulate pre‐mRNA splicing in a cell type‐specific manner [45]. Next, we did not examine that the effect of *Qki* deletion on VSMC plasticity in vivo by specific knockdown or using a Qk^v^ mouse line. A future study should make efforts to clarify the different isoforms of QKI and their effects on VSMC plasticity and vascular remodeling.

Overall, our study identifies cZFP609 as a master regulator in remedying aberrant VSMC plasticity. Modulating the QKI–cZFP609 axis may help propel repair without stenosis therapeutic strategy in vascular injury.

## 4 Materials and Methods

### 4.1 Materials

Information about antibodies and reagents used was listed in Table S1.

### 4.2 Carotid Artery Ligation Models

Mice model of carotid artery ligation was prepared as previously described [2]. In brief, 9–12 weeks old male mice were anesthetized with a mixture of 1.125% isoflurane and oxygen. The left common carotid arteries were ligated using a 6‐0 silk ligature. Before this, AAV9–shcZFP609 (Sequence: GUCUGAAAAGCAAUGAUG U) or AAV9–cZFP609 (1 × 10^12^ vg/mL; HANBIO) was injected into the mice via tail vein for 35 days.

### 4.3 Hematoxylin and Eosin Staining

The left carotid arteries were collected after ligation for 21 days by subjecting mice to 100% CO_2_ inhalation followed by cervical dislocation, fixed, and paraffin‐embedded. Serial sections (5 µm) were prepared at 500 µm proximal from the ligation site for hematoxylin and eosin staining. The ratio of intima and media areas (I/M) were analyzed by a single observer in a blinded manner.

### 4.4 Cell Culture and Treatment

The VSMCs of mouse aortas were isolated and cultured, as previously described [3]. The primary VSMCs grown in low glucose Dulbecco's modified Eagle's medium supplemented with 10% fetal bovine serum to 90% confluence were passaged and identified by α‐SMA positive staining using immunofluorescence. VSMCs of 8–10 passages were used to experiments. VSMCs were treated by normoxic (21% O_2_) or hypoxic (1% O_2_) for 24 h or pretreated with EX527 (20 µM) or RSV (25 µM) for 4 h to inhibit or activate SIRT1 before hypoxia. VSMCs were treated by PDGF‐BB (10 ng/mL) or TNF‐α (10 ng/mL) for the indicated time to induce phenotypic switching.

### 4.5 Analysis of Proliferation and Migration

The proliferation activity of VSMCs was detected by labeling BrdU. Cell‐wounding assay was used to test VSMC migration. The migratory activity was evaluated by the number of cells migrating into the wound area.

### 4.6 siRNA Transfection

The siRNAs targeting mouse cZFP609 (si‐cZFP609), 5′‐GUCUGAAAAGCAAUGA UGUTT‐3′, 5′‐ACAUCAUUGCUUUUCAGACTT‐3′ or QKI (si‐QKI), 5′‐GAGAAUCCUUGGACCUAGATT‐3′ and 5′‐UCUAGGUCCAAGGAUUCUCTT‐3′ were synthesized from GenePharma (Shanghai, China). Control siRNA (si‐Con) 5′‐UUCUCCGAACGUGUCACGUTT‐3′, 5′‐ACGUGACACGUUCGGAGAATT‐3′ was used as a negative control. VSMCs were transfected by the siRNAs by Lipofectamine RNAiMAX Transfection Reagent according to the manufacturer's protocol.

### 4.7 Plasmid Construction and Transfection

Mouse cZFP609 cDNA was provided by Sangon Biotech (Shanghai, China) and cloned into pcD‐ciR vector (Geneseed Biotech, Guangzhou, China) to construct cZFP609 overexpression plasmids.

The sequence of ZFP609 pre‐mRNA deleted QRE2 (‐263−237) or QRE3 (50−107) of QKI binding regions were synthesized and inserted into pcDNA3.1 Vector by Sangon Biotech to construct ZFP609 deletion mutant.

The sequences of WT and the mutants of K132Q, K133Q, and K134Q of QKI were synthesized and inserted into pcDNA3.1 Vector by Sangon Biotech to construct Ac‐mimic mutants (mutation from K to Q). The constructed plasmids were transfected using Lipofectamine 2000 according to the manufacturer's instructions.

### 4.8 Immunofluorescence Analysis

VSMC monolayers were fixed in the acetone, incubated with specific antibodies against PDGFRβ, EEA1, Rab7, or Rab11 at 4°C overnight, and incubated with fluorescein‐conjugated secondary antibodies (Alexa Fluor 488 or Alexa Fluor 555) at room temperature for 1 h. Digitized image data were acquired by a Confocal Laser Scanning Microscope Systems (Leica) and analyzed with the software program LAS AF Lite or Image J [11].

### 4.9 Fluorescence in Situ Hybridization

The VSMCs fixed in 4% paraformaldehyde were permeabilized overnight with 70% ethanol, prehybridized in 50% formamide and 2×SSC and incubated sequentially with Rab7 (1:200) at 4 °C overnight and a secondary antibody (Alexa Fluor 488).

For FISH, VSMCs were incubated with a fluorescence‐labeled specific probe of cZFP609 in a hybridization solution at 37°C for 12 h. DAPI was used to stain the cell nuclei were stained and imaged by a Confocal Laser Scanning Microscope Systems (Leica) [43].

### 4.10 Quantitative Reverse Transcription‐PCR

To quantify the circRNA, total RNAs of mouse arterial tissues and VSMCs were extracted using TRIzol reagent, and 1 µg of RNAs was reverse‐transcribed to cDNA using the M‐MLV First Strand Kit according to the manufacturer's protocol. The primers of mouse cZFP609 were as follow, forward 5′‐GGCCACTAAAGAAAGTCAAGTCTG‐3′ and reverse 5′‐GGACATCTTAGAGTCAACGTCCC‐3′. Quantitative PCR was conducted using SYBR Green qPCR SuperMix‐UDG in duplicate with at least three independent experiments. The relative level of cZFP609 was normalized to β‐actin using the 2^−ΔΔCt^ method.

### 4.11 Western Blotting

The protein lysates (20–60 µg) were separated by 10% sodium dodecyl sulfate–polyacrylamide gel electrophoresis (SDS‐PAGE) gel and then transfered to a polyvinylidene fluoride membrane. The membranes were incubated with the primary antibodies at 4°C overnight, and then with the secondary antibody for 1 h. The protein bands were analyzed with the GE ImageQuant™ LAS 4000 detection system. The antibodies used by the experiments were listed as follows in Table S1.

### 4.12 Immunoprecipitation Assay

The cellular lysates were incubated with 30 µL of Dynabeads protein G, 5 µg of antiacetyl Lysine, anti‐human SIRT1 (hSIRT1), anti‐mouse SIRT1 (mSIRT1) and anti‐PDGFRβ antibodies for 24 h, respectively. The immunoprecipitated proteins were detected by Western blot as described above.

### 4.13 RNA Immunoprecipitation Assay

The cellular lysates were incubated with 5 µg of the primary antibodies at 4°C for 2 h, and then incubated with 50 µL of Dynabeads Protein G at 4°C for 4 h. The pellets were resuspended in 1 mL TRizol Reagent. The quantitative reverse transcription‐PCR (qRT‐PCR) was conducted to detect the immunoprecipitated cZFP609 [11].

### 4.14 RNA Pull‐Down Assay

The cellular lysates were mixed with 3 µg of biotinylated DNA probes against cZFP609, and the binding reaction was conducted by adding 50 µL of Dynabeads™ MyOne™ Streptavidin C1 magnetic beads at 4°C for 2 h. The pulled‐down protein complexes were analyzed by Western blot [43].

### 4.15 Statistical Analysis

Statistical analysis was conducted with SPSS software version 21.0. Differences were assessed by performing one‐way analysis of variance (ANOVA). Comparison between the two groups was conducted by using Student's *t*‐test. Data were generated from at least three independent experiments in triplicates and presented as the means ± SEM. For all statistical comparisons, *p *< 0.05 was considered significant.

## Author Contributions

Mei Han and Fan Zhang designed and supervised experiments. Yong‐Qing Dou, Xiao‐Yun Zhang, and Rui‐Juan Guo conducted most of the experiments. Xiao‐Fu Huang, Yu Song, Xin‐Long Liu, Jie Shi, Fan‐Qin Li, Dan‐Dan Zhang, Peng Kong, Han Li, and Lei Nie assisted in the experiments. Yong‐Qing Dou analyzed data and generated the illustrations. Mei Han edited and revised the manuscript. All authors have read and approved the manuscript.

## Ethics Statement

The Animal Care and Use Committee of Hebei Medical University approved all animal experiments (JACUC‐Hebmu‐P2022005).

## Conflicts of Interest

The authors declare no conflicts of interest.

## Supporting information



Supporting Information

## Data Availability

The data that support the findings of this study are available from the corresponding author upon reasonable request.
