# Supplementary material for: Quaking–cZFP609 Axis Remedies Aberrant Plasticity of Vascular Smooth Muscle Cells via Mediating Platelet‐Derived Growth Factor Receptor β Degradation
Source: MedComm (2020). 2025 Apr 16;6(5):e70167. doi: 10.1002/mco2.70167 (PMC12000678; doi:10.1002/mco2.70167)
Supplement: Supplementary file 1 — Supporting Information [file MCO2-6-e70167-s001.docx]

**Quaking-cZFP609 axis remedies aberrant plasticity of vascular smooth muscle cells via mediating platelet-derived growth factor receptor β degradation**

Yong-Qing Dou^1,4#^, Xiao-Yun Zhang^4#^, Rui-Juan Guo^1,2,3#^, Xiao-Fu Huang^1^, Yu Song^1,2,3^, Xin-Long Liu^1^, Jie Shi^1^, Fan-Qin Li^1^, Dan-Dan Zhang^1,2,3^, Peng Kong^1,2,3^, Lei Nie^1,2,3^, Han Li^5^, Fan Zhang^2,3^*, Mei Han^1,2,3^*

^1^ Department of Biochemistry and Molecular Biology, College of Basic Medicine,

^2^ Key Laboratory of Neural and Vascular Biology of Ministry of Education,

^3^ Key Laboratory of Vascular Biology of Hebei Province, Hebei Medical University, Shijiazhuang, China.

^4^ College of Integrative Medicine, Hebei University of Chinese Medicine, Shijiazhuang, China.

^5^ Department of Orthopaedic Surgery, Institute of Biomechanical Science and Biomechanical Key Laboratory of Hebei Province, Third Hospital of Hebei Medical University, Shijiazhuang, China

^#^These authors contributed equally.

**Correspondence to**

Mei Han, MD, PhD., email: hanmei@hebmu.edu.cn

Fan Zhang, PhD., email: zhangfan86@hebmu.edu.cn

Running title: QKI-cZFP609 axis mediates PDGFRβ degradation

Supplementary Table 1 Detailed information about antibodies and reagents

| Antibodies and reagents | Source | Identifier |
| --- | --- | --- |
| PDGFRβ | Cell Signaling Technology | Cat# 3169 |
| EEA1 | Cell Signaling Technology | Cat# 3288 |
| Rab7 | Cell Signaling Technology | Cat# 9367T |
| Rab11 | Cell Signaling Technology | Cat# 5589 |
| PCNA | WANLEIBIO | Cat# WL03213 |
| OPN | Arigo | Cat# ARG55186 |
| α-SMA | Abcam | Cat# ab32575 |
| SM22α | Abcam | Cat# ab14106 |
| QKI | Abcam | Cat# ab126742 |
| MBL | Abcam | Cat# ab108519 |
| acetyl Lysine | Abcam | Cat# ab22550 |
| Human SIRT1 | Santa Cruz Biotechnology | Cat#sc-15404 |
| Mouse SIRT1 | Millipore | Cat# 07-131 |
| Ubiquitin | Santa Cruz Biotechnology | Cat# sc9133 |
| β-actin | Cell Signaling Technology | Cat# 58169 |
| Ras | Cell Signaling Technology | Cat# 67648 |
| Raf | Affinity | Cat# AF6065 |
| Phosphorylated Raf | Affinity | Cat# AF3064 |
| MEK | Cell Signaling Technology | Cat# 8727T |
| Phosphorylated MEK | Cell Signaling Technology | Cat# 9154T |
| ERK | Cell Signaling Technology | Cat# 4695 |
| Phosphorylated ERK | Cell Signaling Technology | Cat# 4370T |
| Alexa Fluor 488 | Invitrogen | Cat# A-11006 |
| Alexa Fluor 555 | Invitrogen | Cat# A-21428 |
| Prolong Diamond Antifade Mountant with DAPI | Invitrogen | Cat# p36962 |
| Resveratrol | [J&K](http://www.so.com/link?url=http%3A%2F%2Fwww.jkchemical.com%2Findex.aspx&q=J%26K&ts=1471507547&t=1f13a56c45ab9cfcf78a38747d292fb&src=haosou) | Cat# [501-36-0](http://www.jkchemical.com/CH/products/search/fulltextsearch/501-36-0.html) |
| EX527 | Cayman | Cat# 10009798 |
| BrdU Cell Proliferation Assay | Millipore | Cat# 2750 |
| Dynabeads protein G | Invitrogen | Cat# 1004D |
| Streptavidin Dynabeads | Invitrogen | Cat# 65801D |
| DMOG | Sigma | Cat# D3695 |
| First strand cDNA synthesis kit | Beyotime | Cat# D7168M |
| SYBR Green qPCR Mix | Beyotime | Cat# D7265 |
| Lipofectamine 2000 | Invitrogen | Cat# 11668030 |

**
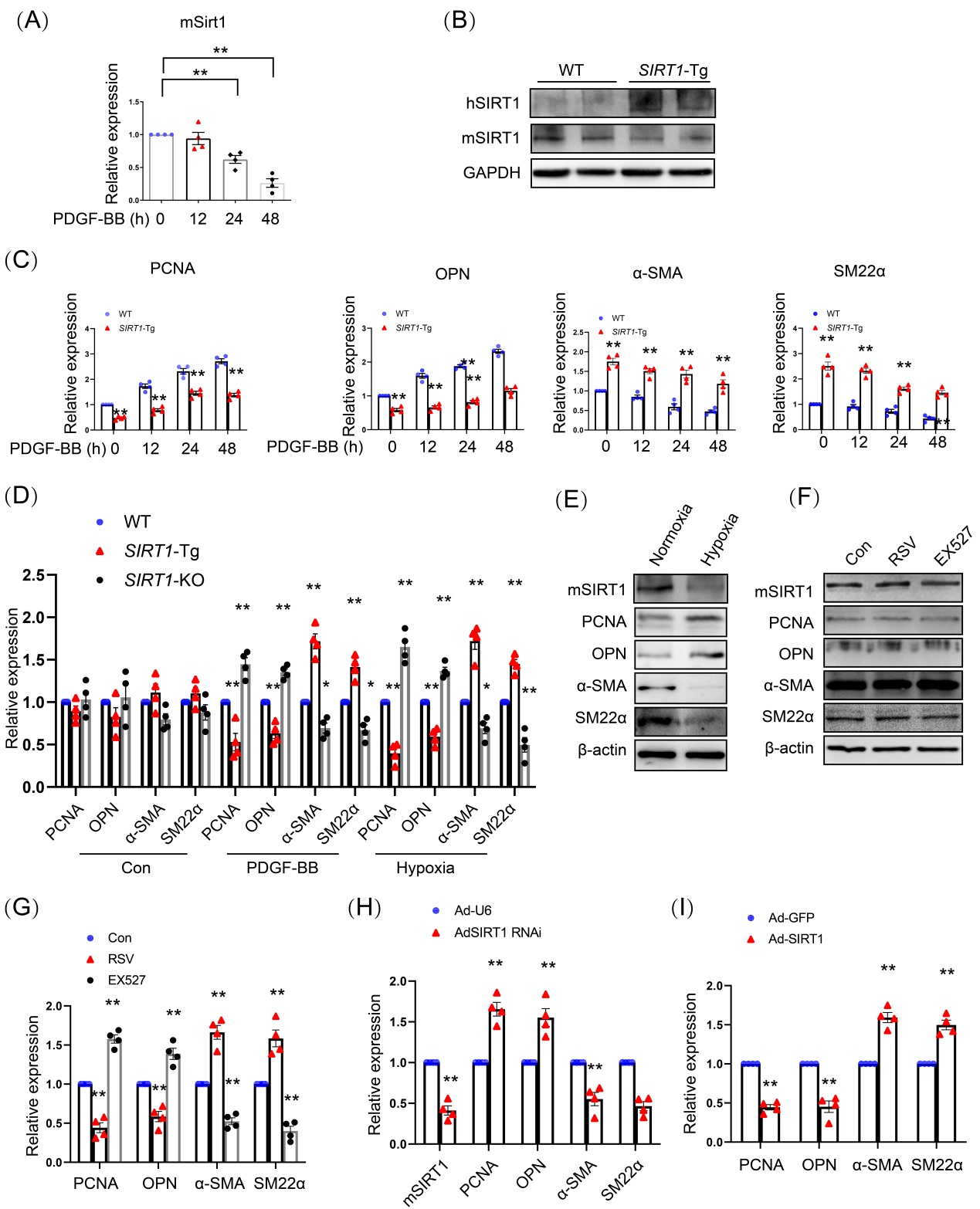
**

SUPPLEMENTARY FIGURE 1. SIRT1 inhibits phenotypic switching of VSMCs. (A, C, D, G-I) Corresponding to FIGURE 1A-F, the graph showed the relative expression of protein/β-actin. (B) Western blot for human SIRT1 (hSIRT1) and mouse SIRT1 (mSIRT1) in WT and *SIRT1*-Tg VSMCs. (E and F) Western blot of mSIRT1, PCNA, OPN, α-SMA and SM22α in WT VSMCs under normoxia and hypoxia (E) or by pretreatment with DMSO, RSV or EX527 for 4 h (F).


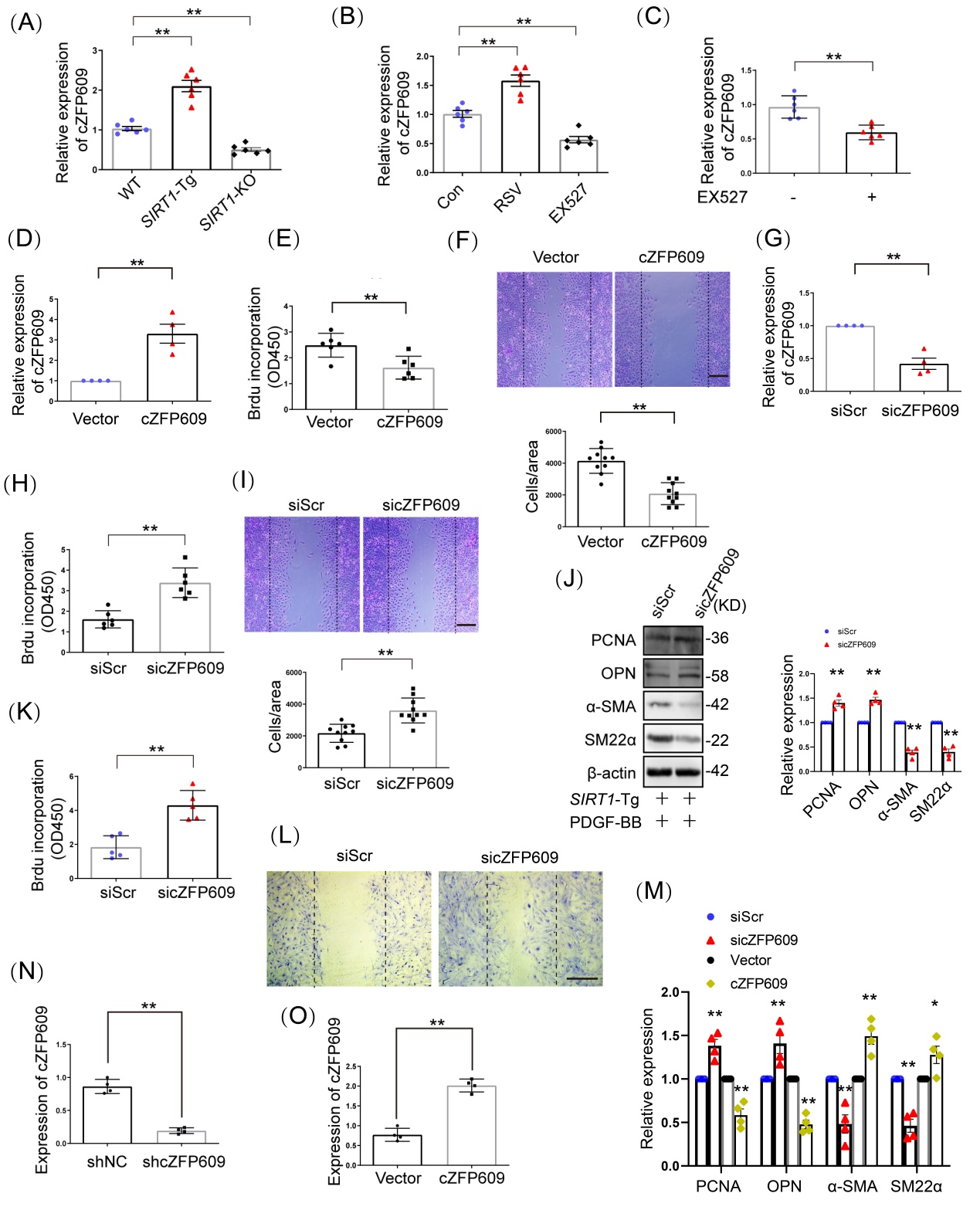


SUPPLEMENTARY FIGURE 2. cZFP609 mediates the inhibitory effect of SIRT1 on phenotypic switching of VSMCs. (A-D, G) qRT-PCRs of cZFP609 expression. (A) In the indicated VSMCs, (B) WT VSMCs pretreated with SIRT1 agonist RSV or inhibitor EX527 for 4 h, (C) *SIRT1-*Tg VSMCs pretreated with EX527 or DMSO for 4 h. (D-F) WT VSMCs were infected by plasmid cZFP609 for 24 h, (D) then exposed to PDGF-BB for 24 h, (E) The relative activity of proliferation by BrdU incorporation in the VSMCs exposed to PDGF-BB for 24 h, (F) The relative activity of migration using a cell-wounding assay in the VSMCs exposed to PDGF-BB for 12 h. Scale bars=100 μm. (G-I) WT VSMCs were infected by sicZFP609 for 24 h, (G) then exposed to PDGF-BB for 24 h, (H) The relative activity of proliferation by BrdU incorporation in the VSMCs exposed to PDGF-BB for 24 h, (I) The relative activity of migration using a cell-wounding assay in the VSMCs exposed to PDGF-BB for 12 h. Scale bars=100 μm. (J-L) *SIRT1-*Tg VSMCs were transfected by sicZFP609 for 24 h, then exposed to PDGF-BB, (J) Western blot of PCNA, OPN, α-SMA, SM22α in the VSMCs exposed to PDGF-BB for 24 h, (K) The relative activity of proliferation by BrdU incorporation in the VSMCs exposed to PDGF-BB for 24 h, (L) The relative activity of migration using a cell-wounding assay in the VSMCs exposed to PDGF-BB for 12 h. Scale bar=200 μm. (M) Corresponding to FIGURE 2F, the graph showed the relative expression of protein/β-actin. (N, O) qRT-PCRs of cZFP609 expression in ligated carotid artery. (N) AAV9-shcZFP609 (1×10^12^ vg /mL, 100 μL per mouse) was injected into *SIRT1*-Tg mice by tail vein for 35 days, then carotid artery ligation for 14 days, (O) AAV9-cZFP609 (1×10^12^ vg /mL, 100 μL per mouse) was injected into *SIRT1*- KO mice by tail vein for 35 days, then carotid artery ligation for 28 days.

SUPPLEMENTARY FIGURE 3. Analysis of KEGG pathway enriched by differentially expressed genes of proteomic analysis between contractile and PDGF-BB-induced synthetic VSMCs.


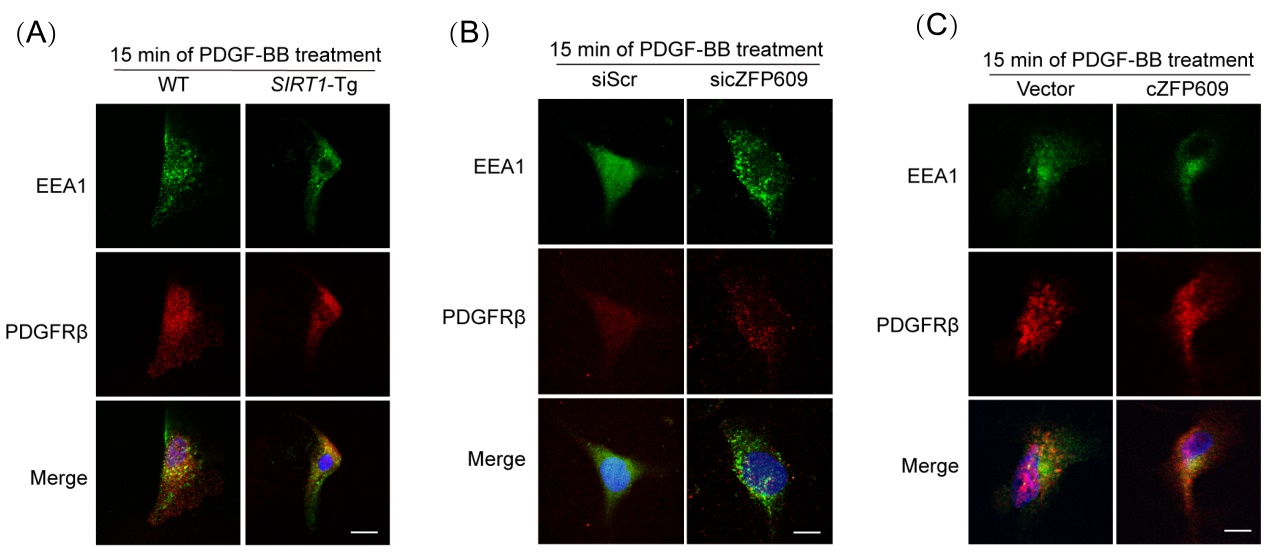


SUPPLEMENTARY FIGURE 4. cZFP609 does not affect the co-localization of PDGFRβ with early endosomal antigen 1 (EEA-1). Co-staining for internalized PDGFRβ (red) and EEA1 (green) in the VSMCs (A), *SIRT1*-Tg VSMCs transfected with siScr or sicZFP609 (B) or *SIRT1*-KO VSMCs transfected by the indicated vectors for 48 h (C) and then exposed to 10 ng/ml of PDGF-BB for 15 min. Scale bars=10 μm.
